# Theory of nonlinear corner states in photonic fractal lattices

**DOI:** 10.1515/nanoph-2023-0443

**Published:** 2023-09-11

**Authors:** Boquan Ren, Yaroslav V. Kartashov, Lukas J. Maczewsky, Marco S. Kirsch, Hongguang Wang, Alexander Szameit, Matthias Heinrich, Yiqi Zhang

**Affiliations:** Key Laboratory for Physical Electronics and Devices, Ministry of Education, School of Electronic Science and Engineering, Xi’an Jiaotong University, Xi’an 710049, China; Institute of Spectroscopy, Russian Academy of Sciences, Troitsk, Moscow, 108840, Russia; Institut für Physik, Universität Rostock, Albert-Einstein-Straße 23, 18059 Rostock, Germany

**Keywords:** higher-order topological insulator, fractal lattice, corner state soliton

## Abstract

We study linear and nonlinear higher-order topological insulators (HOTIs) based on waveguide arrays arranged into Sierpiński gasket and Sierpiński carpet structures, both of which have non-integer effective Hausdorff dimensionality. Such fractal structures possess different discrete rotational symmetries, but both lack transverse periodicity. Their characteristic feature is the existence of multiple internal edges and corners in their optical potential landscape, and the formal absence of an insulating bulk. Nevertheless, we show that a systematic geometric shift of the waveguides in the first generation of such fractal arrays, which affects the coupling strengths between sites of this building block as well as in subsequent structure generations, enables the formation of corner states of topological origin at the outer corners of the array. We find that, in contrast to HOTIs based on periodic arrays, Sierpiński gasket arrays always support topological corner states, irrespective of the direction of the shift of the waveguides, while in Sierpiński carpet structures, corner states emerge only for one direction of the waveguide shift. We also find families of corner solitons bifurcating from linear corner states of fractal structures that remain stable practically in the entire gap in which they form. These corner states can be efficiently excited by injecting Gaussian beams into the outer corner sites of the fractal arrays. Our results pave the way toward the investigation of nonlinear effects in topological insulators with non-integer dimensionality and enrich the variety of higher-order topological states.

## 1 Introduction

Topological insulators are specific materials with insulating bulk that, at the same time, allow currents to flow freely along their edges, where transport is mediated by topologically protected edge states [1, 2]. These unique properties are of great interest as basis for disorder-resistant topological schemes for transmission, routing, and coupling of excitations, as evidenced by ever-growing interest from different areas of physics. Along these lines, topological insulators have been observed in mechanics, acoustics, atomic and optoelectronic systems [3–14] and also on a variety of photonic platforms [15–20]. In particular, in photonics, most of the topological systems reported so far in one-, two-, or three-dimensional settings rely on specially designed *periodic* optical materials possessing topological gaps in the bulk spectrum, see reviews [21–25]. At the same time, the realization of topologically nontrivial photonic systems with *non-periodic* interior and *non-integer effective dimensionality* may substantially expand the realm of practical implementations of topological insulators.

Among such non-periodic topological insulators are recently investigated systems based on *fractal* optical potential landscapes [26, 27], whose complexity and symmetry are determined by the structure of the first-generation element, while the richness of spectrum depends on the order of generation that produces the final structure. Remarkably, these systems can feature hierarchies of multiple internal edges and corners, to the point where they may entirely lack the insulating bulk while still supporting unidirectional protected edge states with broken time-reversal symmetry [27]. The non-integer effective Hausdorff dimensionality of fractal structures is defined as *d*_*f*_ = ln *m*/ln *ℓ*, where *m* determines how many fractal structures of previous generation are needed to construct next generation, while the similarity ratio *ℓ* describes the ratio of geometrical sizes of structures in two subsequent generations. The consequences of this self-similar structure may manifest themselves in unusual diffraction patterns for light beams propagating through them [28], anomalous quantum transport [29], and existence of localized loop linear states in the interior of the structure [30]. A particularly intriguing question is how the non-integer dimensionality of fractal structures correlates with potential possibility to construct on their basis a new type of higher-order topological insulator (HOTI), whose characteristic feature is the existence of topological edge states, with dimensionality at least by 2 lower than dimensionality of the insulator, see recent reports on HOTIs constructed on periodic materials [31–46]. The theoretical link between HOTIs and fractal structures was established only recently using tight-binding models [47, 48], while experimentally fractal HOTIs were studied only in acoustics [49, 50].

Photonic fractal HOTIs remain so far elusive, even in linear case, and the goal of the present work is to introduce them and study their properties in the frames of continuous model accounting for all details of fractal optical potential landscape. Particularly interesting will be to study the impact of nonlinearity on the properties of topological states emerging in such structures. Indeed, nonlinear effects in topological systems [51] may lead to intriguing dynamics of topological excitations, resulting in unusual instabilities [52], resonant phenomena [53], topological transitions [54–57], formation of topological solitons [58–68], and breakdown of topological transport [69–71].

In this work, we explore nonlinear photonic HOTIs realized on Sierpiński gasket and carpet arrays with different discrete rotational symmetries that e.g. can be readily fabricated using the femtosecond-laser direct writing technique in transparent optical materials. To obtain topologically nontrivial structures, we introduce a systematic shift of waveguide positions in the first-generation structures that affects coupling strengths between them. Surprisingly, we find that Sierpiński gasket structure supports topological corner states for any shift of waveguides, as confirmed by calculation of corresponding topological invariant (real-space polarization). This behaviour is in sharp contrast to topological properties of HOTIs with periodic bulk. Fractal HOTI based on Sierpiński carpet instead supports corner states only for specific waveguide shifts. In both structures we have obtained stable corner solitons bifurcating from linear corner states, whose localization properties strongly depend on position of propagation constant in the forbidden gap. Our results may enable the design of corner lasers [72–74] and cavities supporting high-quality modes [75, 76] based on fractal structures, which are crucial for next generation optical functional devices [77–79].

## 2 Results

### 2.1 Theoretical model

**Figure 1:**
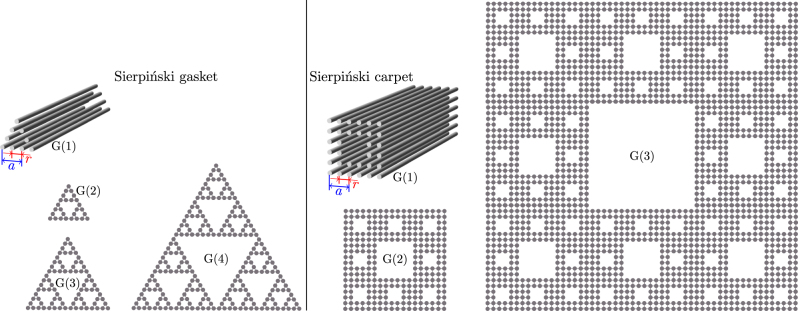
Iterative generation of the Sierpiński gasket and the Sierpiński carpet waveguide arrays. The first generation G(1) of Sierpiński gasket has 9 sites, and the *n*th generation G(*n*) includes three copies of G(*n* − 1). The first generation G(1) of Sierpiński carpet has 32 sites, and the *n*th generation G(*n*) includes eight copies of G(*n* − 1).

The propagation of light in coupled waveguide arrays such as the ones instantiating fractal topological structures can be described by the nonlinear Schrödinger-like equation with focusing cubic nonlinearity

(1) $$i\frac{\partial \psi }{\partial z}=-\frac{1}{2}\left(\frac{\partial^{2}}{\partial x^{2}}+\frac{\partial^{2}}{\partial y^{2}}\right)\psi -R\left(x,y\right)\psi -|\psi {|}^{2}\psi ,$$

where *ψ* is the dimensionless complex amplitude of light field, the transverse coordinates *x*, *y* are normalized to the characteristic scale *r*_0_ = 10 μm, the propagation distance *z* is normalized to the diffraction length $kr_{0}^{2}\approx 1.14\;\;mm$ (corresponding to *z* = 1), *k* = 2π*n*/*λ* is the wave number in the surrounding medium with unperturbed refractive index *n* (for fused silica *n* ≈ 1.45 and the nonlinear refractive index *n*_2_ ≈ 2.7 × 10^−20^ m^2^/W), and *λ* = 800 nm is the design wavelength. The function R(x,y) describes the fractal structure which consists of identical single-mode waveguides placed in the nodes (*x*_*m*_, *y*_*n*_) of the Sierpiński gasket/carpet grid

(2) $$R\left(x,y\right)=\mu \underset{mn}{\sum }e^{-{\left[{\left(x-x_{m}\right)}^{2}+{\left(y-y_{n}\right)}^{2}\right]}^{2}/\sigma^{4}},$$

where the potential depth $\mu =k^{2}r_{0}^{2}\delta n/n$ is proportional to the refractive index contrast *δn* of the array’s waveguides. We further denote the dimensionless width of the waveguides as *σ* = 0.5 (in our normalizations it corresponds to 5 μm) and a default spacing between next-neighbour waveguides along the side of the structure equal to *a* = 3.5 (corresponding to 35 ), see Figure 1. In this work, we choose *μ* = 12.0, equivalent to *δn* ≈ 1.3 × 10^−3^. While these parameters are typical for the femtosecond laser-written waveguides in fused silica [80–83], we would like to note other platforms such as photorefractive SBN crystals [30, 63, 84] and hot atomic vapors [85] may likewise serve as potential experimental testbeds for realization of nonlinear fractal HOTIs.

### 2.2 Linear spectra of fractal arrays

In Figure 1 we display the Sierpiński gasket and carpet fractal waveguide arrays of different generations. As one can see, these structures are self-similar, their effective Hausdorff dimensionality can be defined as *d*_*f*_ = ln 3/ln 2 ≈ 1.585 and *d*_*f*_ = ln 8/ln 3 ≈ 1.893, for Sierpiński gasket and carpet, respectively. Each generation G(*n*) contains *N* = 3^*n*+1^ waveguides for Sierpiński gasket arrays and *N* = 2^3*n*+2^ waveguides for Sierpiński carpet arrays. Further we will consider the fourth generation G(4) of Sierpiński gasket and the second generation G(2) of Sierpiński carpet structures that contain 243 and 256 waveguides, respectively. These fractal arrays (with comparable number of waveguides in selected generations) possess $C_{3}$ and $C_{4}$ discrete rotational symmetry, but lack periodicity. In addition, both structures are characterized by the presence of multiple internal edges and corners, i.e. formally they lack insulating bulk in contrast to usual HOTIs constructed on periodic lattices [45]. We further introduce a shift of waveguides in the first generation G(1) of these structures by varying distance *r*, as indicated in Figure 1 for each type of fractal array. When varying *r*, we keep the spacing between next-neighbour waveguides fixed and equal to *a*. Clearly, the spacing between all nearest-neighbour waveguides becomes identical and equal to 0.5*a* if *r* = 0.5*a*.

To explain the appearance of linear higher-order corner states in fractal arrays and to provide the illustration of transformation of linear spectrum accompanying shift of the waveguides that was not presented in previous literature [48–50], particularly for Sierpiński gasket arrays, we omit the nonlinear term in Eq. (1) and search for linear modes of corresponding fractal optical potentials R in the form *ψ* = *u*(*x*, *y*)*e* ^*ibz*^, where *u*(*x*, *y*) is the real function describing the mode profile that satisfies the equation

(3) $$bu=\frac{1}{2}\left(\frac{\partial^{2}}{\partial x^{2}}+\frac{\partial^{2}}{\partial y^{2}}\right)u+Ru,$$

and *b* is the propagation constant and dimensionless (*b* = 1 corresponds to ≈877.2 m^−1^). This linear problem was solved using the plane-wave expansion method.

**Figure 2:**
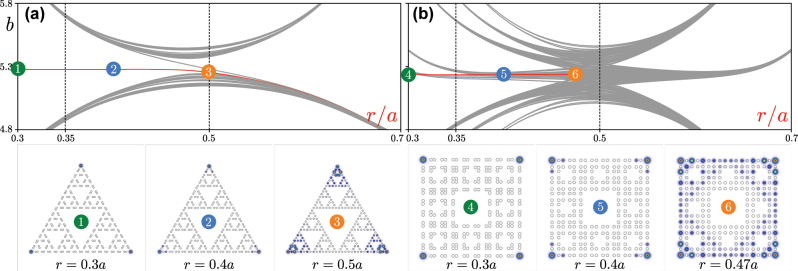
Spectra and states. (a) Linear spectrum of the G(4) Sierpiński gasket waveguide array and examples of corner states for different *r*/*a* values. Field modulus distributions in the bottom row are superimposed on array structure (hollow dots). The modes are shown within the −35 ≤ *x*, *y* ≤ 35 window. (b) Linear spectrum of the G(2) Sierpiński carpet array and examples of its corner modes shown within the window −20 ≤ *x*, *y* ≤ 20. In both panels (a) and (b), red lines correspond to corner modes, while gray lines correspond to various edge and delocalized states.

The dependence of propagation constants *b* of all eigenmodes of G(4) Sierpiński gasket and G(2) Sierpiński carpet arrays on *r* is presented in Figure 2(a) and (b), respectively. One can see that the gradual displacement of waveguides leads to substantial modifications of the spectrum. The propagation constant of corner states in G(4) Sierpiński gasket array is indicated by the red line in Figure 2(a), while gray lines correspond to states extended in at least one direction. At *r* < 0.5*a* corner states form in the gap and are well-isolated from bands of extended states. Remarkably, in this fractal structure, corner states persist even at *r* > 0.5*a* (in Section 2.3 we describe the reason behind this), although in this regime they strongly overlap with the band and show rather weak localization. Due to the $C_{3}$ symmetry of the gasket array, corner states emerge in practically degenerate triplets. Notice that in contrast to HOTIs with similar $C_{3}$ symmetry constructed on usual kagome arrays [86], around *r* = 0.5*a* one can resolve in spectrum two bands of extended states connected by a gray line, also corresponding to extended states.

Representative field modulus distributions in corner states at *r* = 0.3*a*, *r* = 0.4*a* and *r* = 0.5*a*, marked as states 1, 2 and 3, are presented in the bottom row of Figure 2(a). One can see progressively increasing localization in the corners of insulator with decrease of *r*, but even at *r* = 0.5*a* and beyond, the corner state remains localized, in complete contrast to non-fractal kagome HOTIs, where in this limit all states become extended. Remarkably, the localization of the above corner states is virtually unaffected by the generation order *n* of the Sierpiński gasket structure, and such corner states are found in larger structures corresponding to next generations. Moreover, in this structure one does not observe the appearance of localized states in any internal corners—they emerge only in the outer corners.

The linear spectrum of the second generation G(2) of Sierpiński carpet array is depicted in Figure 2(b) as a function of the relative displacement *r*/*a*. It is substantially richer than that of its gasket counterpart, and involves more bands of extended states. It should be stressed that now corner states appear only for one direction of waveguide shift, namely, they can be detected for approximately *r* ≲ 0.47*a*, as indicated by the red line. Interestingly, corner states in carpet fractal structure can overlap with the gray band of extended states, but for above mentioned *r* values, this does not lead to their hybridization with extended states (a similar phenomenon was observed for corner states in the two-dimensional Su-Schrieffer-Heeger model [68]). The propagation constant of corner state shifts into the gap for sufficiently small *r* values, while they are still detectable at *r* ≈ 0.47*a* (for larger *r* values the states extend over the entire structure). Due to $C_{4}$ discrete rotational symmetry of the structure, corner states appear in its spectrum in nearly degenerate quartets. The examples of corner states in Sierpiński carpet array corresponding to numbers 4, 5, and 6 are presented in the bottom row of Figure 2(b).

The robustness of linear corner states can be checked by introducing random perturbations into waveguide positions and/or their refractive index contrast [74]. Numerical simulations show that, for example, in Sierpiński carpet array with *r* = 0.35*a* corner states remain in the gap in the spectrum even for perturbations of waveguide depth *μ* of the order of 10 % that dramatically exceeds the level of possible depth fluctuations in fs-laser written structures.

### 2.3 Topological analysis

The appearance of corner states in fractal arrays can be associated with real-space polarizations [49, 50], which can be written as

(4) $$\begin{array}{c} P_{p}=-\frac{i}{2\pi }\ln\left[\det\left(S_{p}\right)\right],\;S_{p,m,n}=U_{m}^{†}e^{i2\pi \hat{p}/L_{p}}U_{n},\\ P_{q}=-\frac{i}{2\pi }\ln\left[\det\left(S_{q}\right)\right],\;S_{q,m,n}=U_{m}^{†}e^{i2\pi \hat{q}/L_{q}}U_{n}, \end{array}$$

where *L*_*p*_ (*L*_*q*_) is the length of the array along *p* (*q*) direction, $\hat{p}$ $\left(\hat{q}\right)$ is the position operator, *U*_*n*_ is the eigenfunction of *n*th state of the fractal array obtained with periodic boundary conditions in both *p* and *q* directions.

**Figure 3:**
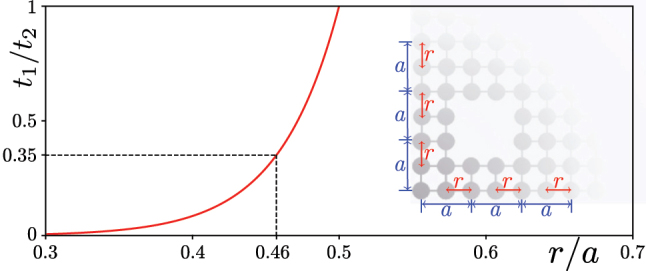
Dependence of the coupling constant ratio *t*_1_/*t*_2_ on the waveguide shift *r*. The notations for coupling constants *t*_1_ and *t*_2_ are presented in the inset, together with *r* and *a* notations.

For G(2) Sierpiński carpet array, *p* and *q* represent *x* and *y*, respectively, and the corresponding topological transition has been discussed previously in the frames of the tight-binding models [49, 50]. For instance, it was shown that if one denotes by *t*_1_ and *t*_2_, respectively, the “intra-cell” and “inter-cell” coupling constants (see the inset in Figure 3 for definition of these constants in our structure), then the real-space polarizations are given by (*P*_*x*_, *P*_*y*_) = (0.5, 0.5) for topologically nontrivial regime that occurs at *t*_1_/*t*_2_ < 0.35 and by (*P*_*x*_, *P*_*y*_) = (0, 0) in non-topological regime at *t*_1_/*t*_2_ > 0.35, if the 1/4 filling is considered. To stress that this is consistent with our results obtained in the continuous photonic model that also takes into account long-distance coupling between all waveguides, in Figure 3 we plotted the dependence of the ratio of coupling constants *t*_1_/*t*_2_ on waveguide shift *r*. The coupling constants were calibrated using two-waveguide structure, since they are determined by the difference of propagation constants |*b*_1_ − *b*_2_|/2 of its two localized modes. Figure 3 shows that the ratio *t*_1_/*t*_2_ = 0.35 corresponds to *r* ≈ 0.46*a* that is consistent with Figure 2(b), where corner states in the carpet structure emerge for *r* ≤ 0.47*a*.

**Figure 4:**
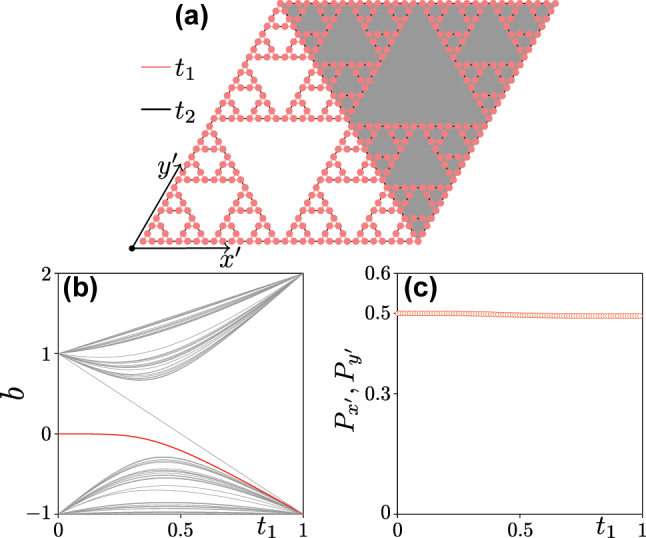
Topological analysis. (a) Composite G(4) Sierpiński gasket array with red bonds corresponding to “intra-cell” coupling constants *t*_1_ and black bonds corresponding to the “inter-cell” coupling constants *t*_2_. (b) Spectrum of the G(4) Sierpiński gasket array obtained using the tight-binding model. (c) Real-space polarizations $\left(P_{x^{\prime }},P_{y^{\prime }}\right)$ versus *t*_1_ with *t*_2_ = 1 − *t*_1_.

Turning to real-space polarizations for the G(4) Sierpiński gasket, array we introduce, for convenience of analysis, an auxiliary array with shaded background to form a composite structure with rhombic landscape, see Figure 4(a) (see also [48]). The Hamiltonian of the system can be written directly using tight-binding approximation and accounting for nearest-neighbor coupling only. Now *p* and *q* directions correspond to *x*′ and *y*′ directions indicated in the figure. Corresponding real-space polarizations are $\left(P_{x^{\prime }},P_{y^{\prime }}\right)$ and taking into account the symmetry of the structure one can conclude that *P*_*x*′_ = *P*_*y*′_. To calculate real-space polarizations, we assume that (i) there are 32 sites in each row along *x*′ (and also in each column along *y*′), vacant places are filled with virtual sites that do not contribute to the Hamiltonian; (ii) we omit the contribution of the sites of the auxiliary array to the Hamiltonian, its sole role is to add corresponding elements into the Hamiltonian matrix. Scanning the value of *t*_1_ from 0 to 1 under the assumption that *t*_2_ = 1 − *t*_1_, one obtains the spectrum of corresponding tight-binding Hamiltonian shown in Figure 4(b), which is consistent with array spectrum in Figure 2(a), obtained in continuous model. In the tight-binding model, the corner state (red curve) is visible in the entire interval 0 ≤ *t*_1_ ≤ 1. Calculating polarizations using Eq. (4) and assuming 1/2 filling (i.e., the band below the corner state is filled), one obtains that real-space polarizations $\left(P_{x^{\prime }},P_{y^{\prime }}\right)=\left(0.5,0.5\right)$ for any *t*_1_, see Figure 4(c), which means that Sierpiński gasket array is always in the topological phase and therefore supports protected corner states – in stark contrast to the behaviour of the carpet structure.

### 2.4 Nonlinear corner states in fractal arrays

Next we turn to nonlinear corner states in fractal arrays. We search for corresponding solutions of Eq. (1) with the nonlinear term included also in the form *ψ* = *u*(*x*, *y*)*e* ^*ibz*^, where now the shape of nonlinear solution *u*(*x*, *y*) corresponding to propagation constant *b* should be found iteratively using Newton’s method. Remarkably, such corner solitons can bifurcate from localized linear corner states supported by corresponding fractal potential landscapes R .

**Figure 5:**
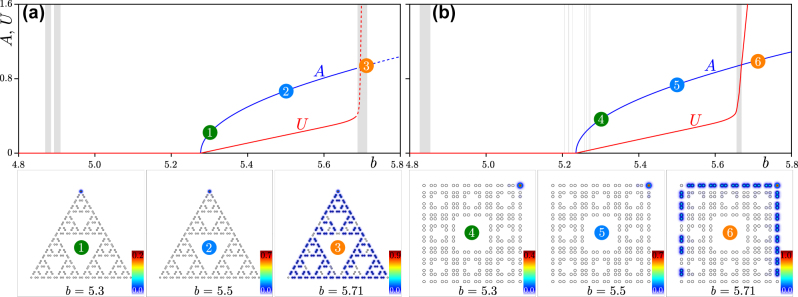
Nonlinear corner state family. (a) Nonlinear corner state family in the G(4) Sierpiński gasket array with *r* = 0.35*a*. The blue and red curves show peak amplitude and power of the nonlinear corner state. Stable branches are shown with solid curves, while unstable branches are shown with dashed curves. Gray regions show bands of extended states. Bottom row shows examples of field modulus distributions corresponding to colored dots in (a). These distributions are superimposed on array profiles shown by hollow circles. (b) Family of corner solitons and examples of their profiles in G(2) Sierpiński carpet array with *r* = 0.35*a*.

The family of corner solitons in the G(4) Sierpiński gasket array is displayed in Figure 5(a), where the dependencies of peak amplitude of the nonlinear corner state *A* = |*ψ*|_max_ and its power *U* = ∬ |*ψ*|^2^d*x*d*y* on propagation constant *b* are shown with blue and red curves, respectively. The dimensionless intensity |*ψ*|^2^ corresponds to the intensity $I=n|\psi {|}^{2}/k^{2}r_{0}^{2}n_{2}$ , where *n*_2_ is the nonlinear refractive index of the material. The relation between the intensity and power *U* is given by [87]

$$\iint IdXdY=\frac{n}{k^{2}n_{2}}\iint |\psi {|}^{2}dxdy=\frac{n}{k^{2}n_{2}}U,$$

where *X*, *Y* are real dimensional coordinates. Here, for illustrative purposes, we consider the linear corner states of a structure with *r* = 0.35*a*, and the well-localized solitons bifurcating from them [see solutions 1 and 2 in the bottom row of Figure 5(a)]. In contrast to the usual behaviour of lattice solitons that become more localized with increase of power, when propagation constant of corner soliton in fractal array approaches the band of extended states, its power starts increasing rapidly, but one observes abrupt delocalization, due to coupling with extended states [see solution 3 in the bottom row of Figure 5(a) that penetrated into the band]. Notice also that nonlinearity localizes light in one corner only and that corner solitons in fractal structure are thresholdless since they bifurcate from linear topological states.

The family of nonlinear corner state in the G(2) Sierpiński carpet likewise bifurcates from corresponding linear corner states and is shown in Figure 5(b) for *r* = 0.35*a*. Representative examples of nonlinear corner states numbered 4, 5 and 6 are shown in the bottom row of Figure 5(b). For this value of *r*, linear corner states have propagation constants in the gap, they are well-localized and give rise to similarly well-localized corner solitons, see states 4 and 5. Notice that even though propagation constant of such soliton crosses one of the bands soon after bifurcation, it does not couple with corresponding extended states. Instead, the expansion occurs only when soliton enters into next band [see state numbered 6 in the bottom row of Figure 5(b)] as the corner soliton hybridizes mainly with edge states at the outer edge.

The stability of the nonlinear corner states in both G(4) Sierpiński gasket and G(2) Sierpiński carpet arrays was subsequently studied by adding a small-scale perturbation (5 % in amplitude) into them and propagating them in the frames of Eq. (1) over a very long distance *z* ≈ 4000 that by several orders of magnitude exceeds typically available sample lengths (for fs-second laser direct written waveguide arrays, the sample length of 10 cm corresponds to a dimensionless propagation distance *z* ≈ 88 with diffraction length of $kr_{0}^{2}\approx 1.14\;\;mm$ used for normalization). In Figure 5(a) and (b) stable corner state families are shown by solid curves, while unstable families are shown dashed. One can see that for our parameters solitons destabilize when they enter the band in gasket array, and they are always stable in carpet structure. This illustrates that nonlinear corner states in fractal arrays are rather robust objects in a broad range of peak amplitudes.

### 2.5 Excitation of corner states in fractal arrays

**Figure 6:**
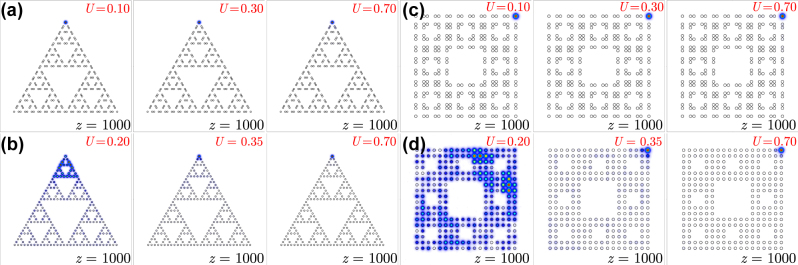
Output field modulus distributions at *z* = 1000 for Gaussian excitations of corner waveguide in G(4) Sierpiński gasket arrays with *r* = 0.35*a* (a) and *r* = 0.50*a* (b), and in G(2) Sierpiński carpet arrays with *r* = 0.35*a* (c) and *r* = 0.50*a* (d). Input power levels are indicated on the plots.

To demonstrate the possibility of dynamical excitation of the nonlinear corner states reported in Figure 5, we simulate Gaussian beams with different powers launched into the corner waveguide and we follow their propagation dynamics up to the distance *z* ≈ 1000. In Figure 6(a), we show the output field modulus distributions for different input powers in G(4) Sierpiński gasket array with *r* = 0.35*a*. In this case, the output remains well localized for all considered power levels indication on corner soliton formation. Similar results were obtained for G(2) Sierpiński carpet array in topological phase at *r* = 0.35*a*, as shown in Figure 6(c). In G(4) Sierpiński gasket array with *r* = 0.50*a* the dynamics is rather different. Owing to the substantially weaker localization of corner states that are still present in linear spectrum in this structure, Gaussian input beam may show moderate expansion at low powers *U* ≈ 0.2, see Figure 6(b). However, further increase of input power leads to a pronounced contraction of light in the excited waveguide. Notice that at even higher input powers *U* > 0.7, the excited state may have a propagation constant in the semi-infinite gap in the spectrum. The G(2) Sierpiński carpet array at *r* = 0.50*a* considered in Figure 6(d) is in the trivial insulator phase and therefore does not support corner states in the spectrum. Accordingly, at low powers, one observes a strong expansion of the input beam over the entire array. Even at *U* = 0.35, when purely nonlinear localization in the corner appears, one still observes strong background inside the array. Finally, at *U* ≈ 0.7, a non-topological corner soliton forms that is similar to conventional lattice corner solitons.

## 3 Conclusions

Summarizing, we have theoretically studied photonic nonlinear fractal HOTIs based on the Sierpiński gasket and Sierpiński carpet waveguide arrays. We have shown that, despite the resemblances in their self-similar structure, these two systems behave rather differently upon introduction of systematic shift of waveguides into first generation structure. While Sierpiński gasket array appears to be in topological phase for any shift of the waveguides, which strongly contrasts with the behaviour of HOTIs based on periodic lattices, its carpet counterpart enters topological phase only for one direction of displacement. We have connected the differences in behaviour of these two systems with real-space polarizations describing their topological properties. In the presence of nonlinearity, both these structures support remarkably stable corner solitons bifurcating from topological corner modes. Our results indicate the possibility of realizing higher-order topological phases in photonic fractal structures with non-integer effective dimensionality, and illustrate the impact of Kerr nonlinearity on light localization in these systems.
